# Discrepancies in Non-Mohs Micrographic Surgery for Non-melanoma Skin Cancer Between Lighter-Skinned and Darker-Skinned Patients

**DOI:** 10.7759/cureus.54027

**Published:** 2024-02-11

**Authors:** Luis J Borda, Iain Noel M Encarnacion, Ryan C Saal, H. William Higgins II, Robert J Pariser

**Affiliations:** 1 Department of Dermatology, Eastern Virginia Medical School, Norfolk, USA; 2 Department of Dermatology, University of Pennsylvania, Philadelphia, USA

**Keywords:** time-to-diagnosis, darker-skinned, squamous cell carcinoma, basal cell carcinoma, non-melanoma skin cancer

## Abstract

Background: Non-melanoma skin cancer (NMSC) is highly prevalent in the United States, with darker-skinned patients (DSP) exhibiting lower incidence but increased morbidity and mortality. The purpose of this study is to elucidate NMSC disparities between DSP (Fitzpatrick skin phototype IV or more) and lighter-skinned patients (LSP, Fitzpatrick skin phototype III or less), focusing on surgical features of non-Mohs micrographic surgery-treated NMSC.

Methods: This retrospective cohort study included LSP and DSP diagnosed with either basal cell carcinoma (BCC) or squamous cell carcinoma (SCC) in an academic dermatology setting. Variables collected included age, gender, type of NMSC, location, staging, time-to-diagnosis (TTD), pre-operative lesion size, and post-operative defect size. Categorical variables were reported as counts and percentages, while the association between categorical variables was assessed using a two-tailed Fisher’s test. A paired t-test was used to determine the association between continuous variables. P-values <0.05 were considered statistically significant.

Results: A total of 27 patients with NMSC were identified, of which 9 (33.3%) were DSP. Patients of darker skin were predominantly female (n=7; 77.8%), while no gender predilection was found in LSP (n=9; 50.0% female; p=0.23). Time-to-diagnosis was significantly longer in DSP than in LSP (61.3 weeks vs 25.1 weeks, respectively; p = 0.02). Despite this, there was no statistical difference in terms of staging, pre-operative lesion size (11.89 mm in DSP vs 10.76 mm in LSP, p=0.75), and post-operative defect size (45.56 ± 29.21 mm in DSP vs 31.22 ± 19.60 mm in LSP; p=0.33).

Conclusions: Darker-skinned patients had a longer TTD without staging differences. Our study confirms the need for reducing TTDs for NMSC in DSP. Action initiatives include continued educational efforts to increase awareness of NMSC risk in DSP and more rigorous routine skin cancer screening.

## Introduction

Non-melanoma skin cancer (NMSC) is the most common malignancy in the United States; however, darker-skinned patients (DSP) are affected at lower rates compared to their lighter-skinned counterparts. Despite this lower incidence, DSP experience increased morbidity and mortality rates, particularly with melanoma [1]. The disparity of insufficient awareness, diagnosis occurring at an advanced stage, or socioeconomic considerations contribute to the overall higher mortality associated with skin cancer diagnosis in this population [1]. The purpose of this study is to highlight the disparities in NMSC between DSP (i.e., skin of color or ethnic skin; Fitzpatrick skin phototype IV or more was used in our study due to objectivity) and lighter-skinned patients (LSP, Fitzpatrick skin phototype III or less) by comparing several surgical features (e.g., surgical defects, time-to-diagnosis (TTD)) of non-Mohs micrographic surgery (MMS)-treated (i.e., wide local excision or electrodessication and curettage) NMSC, namely basal cell carcinoma (BCC) and squamous cell carcinoma (SCC).

## Materials and methods

**Figure 1:**
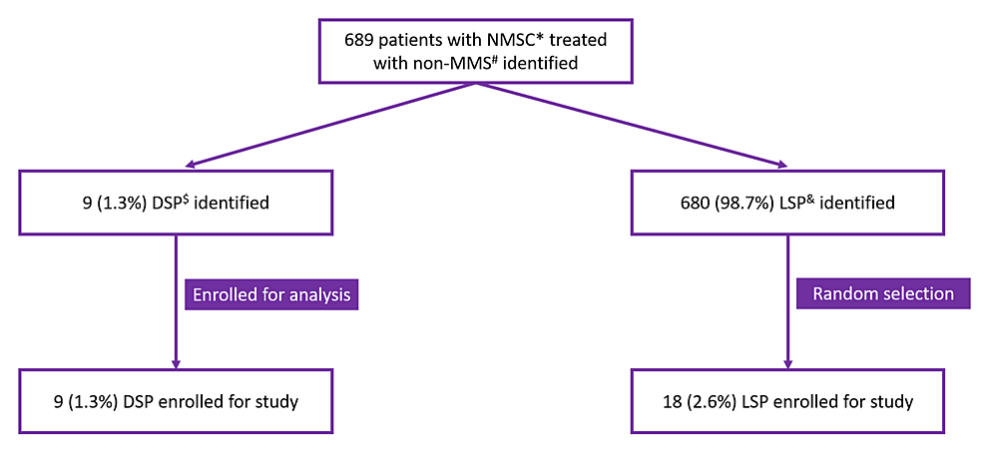
Patients’ selection process A total of 689 patients with NMSC treated with non-MMS were identified. A DSP:LSP ratio of 1:2 was used to assess associations and differences between these two groups. The control group of LSP was randomly selected using the online software Gigacalculator-Random Picker. ^$^DSP: Darker-skinned patients; ^&^LSP: Lighter-skinned patients; *NMSC: Non-melanoma skin cancer; ^#^MMS: Mohs micrographic surgery.

We conducted an Institutional Review Board-approved retrospective study that included LSP and DSP diagnosed with either BCC or SCC in an academic dermatology setting from January 1, 2015, to December 31, 2021. Data from Electronic Medical Records (EMR) were obtained by two independent reviewers, and their accuracy was confirmed by a third reviewer, ensuring inter-rater reliability. Patients' assignments to both DSP and LSP categories were based on the evaluation of clinical photos by the reviewers in the same fashion. The clinical photos stored in the EMR were taken immediately before the diagnostic biopsy was performed. A total of 689 patients with NMSC who underwent non-MMS were identified, of which 1.3% (n=9) were DSP. A DSP:LSP ratio of 1:2 was utilized to assess associations and differences between the two groups. A control group of LSP was randomly selected using the online software Gigacalculator-Random Picker (Figure 1). Variables collected from the patients' EMR included age, gender, type of NMSC, location, staging, TTD (i.e., the time between the initial manifestation of the lesion and the moment at which the lesion underwent diagnostic biopsy), pre-operative lesion size, and post-operative defect size. Categorical variables were reported as counts and percentages, while the association between categorical variables was assessed using a two-tailed Fisher's test. A paired t-test was used to determine the association between continuous variables. P-values less than 0.05 were considered statistically significant.

## Results

A total of 27 patients with NMSC were identified, of which nine (33.3%) were DSP. Lighter-skinned and darker-skinned patients did not differ by age (mean age 64.4 ± 14.19 years and 72.5 ± 11.40 years, respectively; p=0.38). Patients of darker skin were predominantly female (n=7; 77.8%), while no gender predilection was found in LSP (n=9; 50.0% female; p=0.23, Table 1). There was no predisposition for a certain type of NMSC between the two groups (BCC: n=4; 44.5% of DSP vs. n=11; 61.1% of LSP; SCC: n=5; 55.5% of DSP vs. n=7; 38.9% of LSP; p=0.44). Time-to-diagnosis was significantly longer in DSP than LSP (61.3 weeks vs. 25.1 weeks, respectively; p=0.02). Despite this, there was no statistical difference in terms of staging (per Brigham and Women’s Hospital Staging [2,3]; p=0.44) and pre-operative lesion size (mean size 11.89 mm in DSP vs. 10.76 mm in LSP, p=0.75). Although the post-operative defect size tended to be larger in patients of darker skin, the difference was not statistically significant (mean size 45.56 ± 29.21 mm in DSP vs. 31.22 ± 19.60 mm in LSP; p=0.33).

**Table 1:** Demographic and clinical features of patients with NMSC treated by non-Mohs micrographic surgery in LSP and DSP *P-values < 0.05 were considered statistically significant. ^High-risk features per Brigham and Women’s Hospital Staging for cutaneous SCC: clinical tumor diameter ≥ 2 cm, tumor invasion beyond subcutaneous fat, poorly differentiated histology, perineural invasion of nerves ≥ 0.1 mm in caliber. For BCC, tumor diameter ≥ 4 cm, head or neck location, and depth beyond fat. ^α^Length (higher value compared to width) was chosen for comparison. NMSC: non-melanoma skin cancer, LSP: lighter-skinned patients (Fitzpatrick skin phototype III or less), DSP: darker-skinned patients (Fitzpatrick skin phototype IV or more), BCC: basal cell carcinoma, SCC: squamous cell carcinoma.

Characteristic	LSP (n = 18)	DSP (n = 9)	p-value*
Age, years (mean (SD))	72.5 (11.4)	64.4 (14.1)	0.38
Sex (n (%))			0.23
Male	9 (50.0)	2 (22.2)	
Female	9 (50.0)	7 (77.8)	
NMSC type (n (%))			0.44
BCC	11 (61.1)	4 (44.5)	
SCC	7 (38.9)	5 (55.5)	
Time to diagnosis, weeks (mean (SD))	25.1 (29.0)	61.3 (34.6)	0.02
High-risk factors^ (n (%))	4 (22.2)	0 (0)	0.27
Pre-operative lesion size^α^, mm (mean (SD))	10.76 (4.33)	11.89 (6.99)	0.75
Post-operative defect size^α^, mm (mean (SD))	31.22 (19.6)	45.56 (29.21)	0.33

## Discussion

While DSP also face the possibility of developing NMSC, the predominant focus of skin cancer research has been on LSP. Skin cancers observed in DSP frequently exhibit increased morbidity (e.g., surgical outcomes) and mortality rates, potentially attributable to different factors, such as greater loss of health coverage, education level, socioeconomic background, and overall skin cancer risk perceptions [1].

In this study, DSP had a longer TTD than LSP. Similarly, *Tripathi et al.* found that black patients had a longer time from diagnosis to definitive surgery for stages I to III melanoma [4]. Extended TTDs in DSP with NMSC could be a contributing factor to racial variations in NMSC survival, in conjunction with later stage at presentation, biological differences (e.g., genomic and melanin-related differences, UV-induced epigenetic and transcriptomic alterations) in NMSC features [5], and disparities in healthcare utilization. Furthermore, Black, Hispanic, and Asian patients often hold cultural perceptions that suggest that their ethnic backgrounds have little to no risk of developing skin cancer due to their darker skin tone [6]. Additionally, ethnic minorities obtain full-body skin examinations at lower rates compared to White patients [6]. These disparities may be reflected in our data since of the 689 patients identified in this study over a six-year period, only 1.3% were DSP.

Our study revealed that despite longer TTDs in DSP, staging did not differ between both groups. Multiple reports have suggested that NMSCs are often identified at more advanced stages in individuals of Black ethnicity when compared to those of White descent [7]; however, it is important to underscore that a high proportion of advanced cases are referred to tertiary cancer centers; as such, these institutions are more likely to receive more advanced cases of NMSCs. Our sample size could also be a contributing factor to these findings.

We used post-operative defect size as an approximation of carcinoma size. Although the mean pre-operative lesion and post-operative defect sizes were larger in DSP compared to LSP, these differences were not statistically significant. It is pertinent to mention that *Blumenthal et al.* noted that Hispanic/Latino patients exhibited significantly larger MMS-treated NMSC defects compared to their White counterparts [1]. The identification of both larger pre-operative lesion and post-operative defect sizes holds significant importance as it serves as an additional indicator of health disparities rooted in race/ethnicity. Moreover, the presence of larger pre-operative lesion and post-operative defect sizes may be reflective of more advanced disease stages and may be linked to increased morbidity. This observation is likely influenced by various factors. It is essential to acknowledge that challenges in accurately diagnosing NMSC in individuals with darker skin tones are an important factor. This challenge encompasses both clinical and dermatoscopic aspects, where the nuances of skin lesions in DSP may pose difficulties for dermatologists, potentially affecting the precision of diagnosis [6,7]. Additionally, DSP may lack awareness of their susceptibility to skin cancer, leading to reduced adoption of photoprotection or sun-avoidant behaviors, diminished ability to recognize potential skin cancers, and delays in seeking medical attention. *Buster et al. *revealed that Hispanics and Blacks often perceive that skin cancer is preceded by pain or other symptoms, and may feel overwhelmed by numerous skin cancer prevention recommendations [8]. Moreover, according to research conducted by *Taylor*, individuals of Hispanic and Black descent disclosed limited usage of sunscreen, attributing this behavior to misconceptions that render sun protection unnecessary [9]. Finally, the increased sizes of these surgical defects may pose cosmetic challenges, particularly for DSP who already face heightened susceptibility to post-inflammatory hyperpigmentation and scarring.

Limitations of this study include its retrospective design, small sample size, the varying ethnicities of DSP (which limited the ability to conduct more granular analyses among different ethnic groups), and lack of data in many patients’ EMR.

## Conclusions

This study underscores significant disparities in NMSC between DSP and LSP, despite the lower incidence of NMSC among DSP. Our findings reveal that DSP experience longer TTDs compared to LSP, potentially contributing to increased morbidity and mortality rates. While staging, pre-operative lesion size, and post-operative defect size did not significantly differ between groups, we emphasize the potential delays in seeking medical attention and disparities in healthcare utilization shown in other studies. Cultural perceptions, lack of awareness, and cosmetic challenges further compound these disparities. Action initiatives include continued educational efforts to enhance understanding of NMSC risk in DSP as well as advocating for more rigorous routine skin cancer screening in these patients. However, limitations including a small sample size and varied ethnicities among DSP underscore the need for larger, more comprehensive studies to address these inequalities and improve outcomes for DSP affected by NMSC.
